# Evidence of Rapid Evolution in Herbivory Defense Responses With Conserved Trade‐Offs in Populations of *Medicago polymorpha*

**DOI:** 10.1002/ece3.72220

**Published:** 2025-09-25

**Authors:** Shawna L. Rowe, Zoie C. Lopez, Danaka Ross, Cynthia Sackos, Stephanie S. Porter, Maren L. Friesen, Chandra N. Jack

**Affiliations:** ^1^ Department of Plant Biology Michigan State University East Lansing Michigan USA; ^2^ Department of Plant Pathology Washington State University Pullman Washington USA; ^3^ School of Biological Sciences Washington State University–Vancouver Vancouver Washington USA; ^4^ Biology Department Clark University Worcester Massachusetts USA

**Keywords:** biological invasion, plant‐herbivore interactions, rapid evolution, trade‐offs

## Abstract

Theories explaining the evolution of plant defensive strategies are difficult to experimentally test. Biological invasion scenarios can serve as helpful natural experiments for examining the evolutionary dynamics of plant defenses when plants become established as potential hosts in new environments. This study uses a historical invasion by *Medicago polymorpha* (Burr Clover) to test the predictive power of the Shifting Defense Hypothesis (SDH) by investigating variation in plant defenses to herbivorous insects. We compared the feeding preferences of a generalist and a specialist herbivore on native and invasive populations of *M. polymorpha* . We document a shift in herbivore preference patterns for constitutive versus herbivore‐induced tissues when comparing plants from native and invaded ranges. However, specific biochemical defenses showed a conserved negative correlation between constitutive and inducible defenses across both ranges, indicating a fundamental trade‐off in defense strategy that persists despite allocation differences, suggesting defense evolution that was not revealed by tests in this study. These results provide evidence of evolutionary shifts in plant palatability that are consistent with predictions of the SDH, which predicts evolutionary shifts in defense allocation. Our findings reveal complex evolutionary dynamics that underlie invasion success and demonstrate that invasive *M. polymorpha* have undergone evolutionary adaptation in defense strategy beyond any immediate ecological advantages of enemy release, providing insight into how invasive plants successfully adapt to novel herbivore communities over time.

## Graphical abstract

This study uses a historical invasion by *Medicago polymorpha* (Burr Clover) to test the predictive power of the Shifting Defense Hypothesis (SDH) by investigating variation in plant defenses to herbivorous insects. We compared the feeding preferences of a generalist and a specialist herbivore on native and invasive populations of *M. polymorpha* and investigated variation in biochemical defense strategies to explore adaptations resulting from herbivore co‐occurrence in the introduced range. Our findings reveal complex evolutionary dynamics that underlie invasion success and demonstrate that invasive *M. polymorpha* have undergone evolutionary adaptation in defense strategy beyond any immediate ecological advantages of enemy release, providing insight into how invasive plants successfully adapt to novel herbivore communities over time.

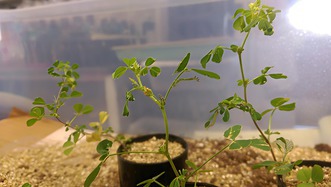

## 1 Introduction

Rapid evolutionary trajectories of invasive plants in novel environments represent a compelling natural experiment in adaptation. When introduced to new habitats, plants and other species can undergo remarkably swift evolutionary changes as they respond to selection pressures that differ from those of their native environments. These changes may stem from differences in biotic stress due to novel enemy and pathogen pressures (Baker 1974; Blossey and Notzold 1995; Bossdorf and Auge 2005) or changes in abiotic stresses such as differences in water availability or soil salinity (Richards et al. 2008; Ozaslan et al. 2016). In novel environments, plants lack the adaptive and co‐evolutionary advantages they may have gained over time in their native habitat (Donoghue and Edwards 2014; Chakraborty et al. 2023). The absence of long‐familiar co‐evolved species paired with novel interactions generates unique selective pressures that drive these quick adaptive changes. The resulting rapid evolutionary responses have consequences that ripple through invaded communities around the world and have implications for ecosystems and the agricultural communities they support (Zangerl and Rutledge 1996; Blossey and Hunt‐Joshi 2003; Agrawal and Hastings 2012). Documenting and understanding the mechanisms driving these periods of accelerated evolution provide critical insight into both invasion dynamics and fundamental evolutionary processes (Broennimann and Guisan 2008; Elliott‐Graves 2016; Novoa et al. 2020).

Complex relationships between plants and insects are of particular interest when investigating rapid evolutionary changes (Baker 1974; Agrawal and Hastings 2012; War et al. 2018; McCulloch and Waters 2023). Novel selection pressures arising during invasions may trigger plants to reallocate resources, such as those involved in anti‐herbivore defenses (Koricheva et al. 2004; Joshi and Vrieling 2005). One study found that common evening‐primrose ( *Oenothera biennis* ) demonstrated reductions in host chemical defenses after only 5 years of pesticide treatments (Agrawal and Hastings 2012). Similarly, insect populations have continuously been shown to undergo rapid evolutionary changes within years of dramatic environmental changes (Garnas 2018), often in less time than plants (Pélissié et al. 2022; McCulloch and Waters 2023). The degree of shared evolutionary history between interacting plant and insect populations often determines the trajectory and pace of these adaptations. Rather than simply categorizing plants as “native” or “invasive,” examining the evolutionary familiarity between interacting species provides deeper insight into ecological outcomes. This perspective becomes increasingly important as the world experiences climate‐driven range shifts (Thuiller 2004).

Numerous competing theories have aimed to predict the likelihood of success for an invading population in the face of threats from novel predators (Elliott‐Graves 2016). Arguably the most prevalent ecological theory, the Enemy Release Hypothesis (ERH), predicts that introduced plants experience reduced regulation from herbivores and pathogens in their novel environment compared to their native ranges. Plants facing herbivores in novel environments will face generalists as a consequence of escaping the environment of their co‐evolved predators—a key assumption for hypotheses such as the ERH (Maron and Vilà 2001). By extension, introduced plants will experience reduced regulation from their historically specialized herbivores and pathogens in their novel environment compared to in their native range. ERH posits that this release from co‐evolved enemies provides an immediate ecological advantage through reduced tissue damage and resource allocation to defense, contributing to enhanced growth and reproductive output (Keane and Crawley 2002; Joshi and Vrieling 2005). While ERH explains immediate ecological advantages following introduction, it does not directly address the evolutionary changes that may occur in plant populations as they adapt to novel herbivore communities over time.

The Shifting Defense Hypothesis (SDH) focuses on adaptive evolutionary changes, suggesting that plants in their native range have evolved costly basal defenses to deter herbivores with which they share long histories (Yi et al. 2024). Evolutionary investment in basal defenses, commonly known as constitutive defenses, can be beneficial when a species is under persistent threat from a nearly constant predator. In contrast to constitutive defenses, plants can conserve resources by deploying induced defenses in response to the presence of a predatory herbivore (Mithöfer and Boland 2012). Upon invading a new range, herbivores that have developed tailored counter‐measures and are thus able to feed on coevolved hosts are typically absent or less abundant; meanwhile, generalist herbivores that feed on any available host consequently become more prevalent predators (Yi et al. 2024). Evidence for differences between plant populations in either or both of these broad classes of herbivore defenses would suggest some degree of evolutionary change in a population's defensive strategy. However, empirical support for the SDH is mixed; some studies have reported invasive populations that show no directional shift toward greater generalist‐targeted or inducible defenses, or clear shifts at all, rather than the change predicted by SDH (Doorduin and Vrieling 2011; Brandenburger and Kim 2020; Yi et al. 2024).

The ERH and SDH hypotheses provide complementary frameworks for conceptualizing how invasive plants adapt to new environments. The ERH focuses on contemporary ecological interactions between plants and their herbivorous enemies, while the SDH focuses on genetic and phenotypic changes in the invading plants many generations after introduction. These frameworks likely operate along a temporal continuum during the invasion process. Plants may experience immediate advantages through enemy release (ERH), with reduced herbivore pressure that ultimately enables rapid population expansion. As invasive populations persist over multiple generations, selection pressures from the novel herbivore community, typically dominated by generalists rather than specialists, drive evolutionary adaptations in defense strategy (SDH). This temporal progression from ecological release to evolutionary optimization may help explain the successful establishment and spread of many invasive plant species over time. Together, these hypotheses highlight the dynamic nature of plant adaptation in response to the novel environmental pressures encountered in invaded ranges.

**FIGURE 1:**
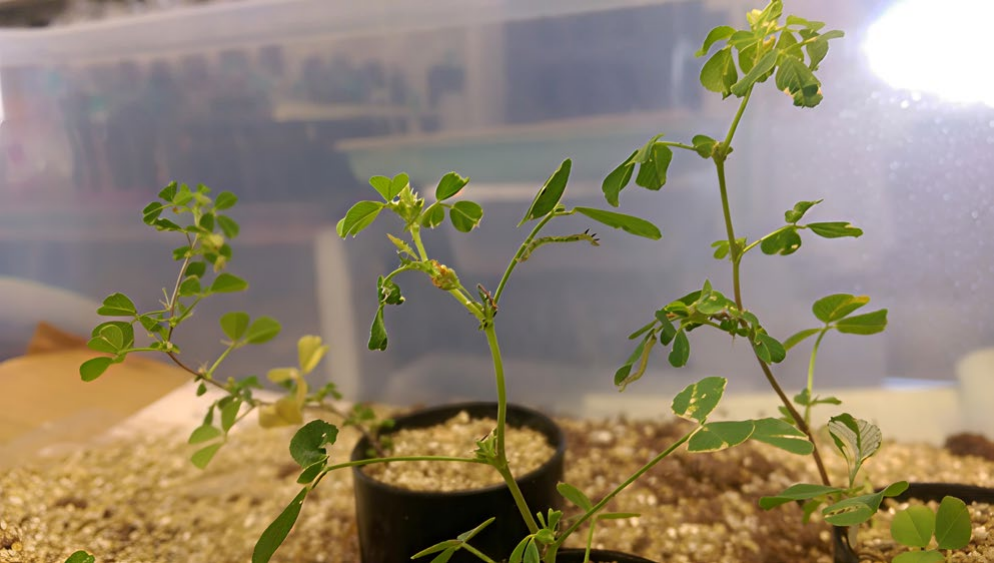
Study system host plant *Medicago polymorpha* and a Soybean Looper caterpillar. Three *M. polymorpha* plants grown in conetainers and placed in a plastic box are shown during their vegetative phases, such as was used in this study. A single Soybean Looper caterpillar is shown on the central *M. polymorpha* plant.

For testing hypotheses of herbivory‐induced population differences, the annual legume Burr Clover ( *Medicago polymorpha* ) serves as an excellent model system (Figure 1). Having migrated from its native Mediterranean range to the Americas nearly 500 years ago via European colonizers (Helliwell et al. 2018; Jack and Friesen 2019), *M. polymorpha* provides an opportunity for a backwards look at adaptation because of the co‐evolutionary history between populations of the invaded range and the local herbivores. Well‐established populations exist throughout the invaded range and are found in both North and South America (Helliwell et al. 2018). In contrast, native range Eurasian *M. polymorpha* is an unfamiliar, or naïve, population to New World herbivores. Previous work on *M. polymorpha* has provided evidence of rapid evolutionary changes including the emergence of a latitudinal clinal variation in the flowering times across its American ranges (Helliwell et al. 2018), leaf size and marking phenotypic variation throughout its invaded Australian range (Lloyd et al. 2025), and evidence of greater fecundity and belowground energy investment across the invaded ranges relative to the native range (Hoffbeck and terHorst 2022). These successful establishments around the globe and the wide distribution throughout the Americas suggest invasive populations have developed effective strategies to deal with local biotic threats.

Testing evolutionary hypotheses like the SDH requires measuring specific defensive traits that may have shifted during the invasion process. While the ERH primarily predicts changes in herbivore preference and damage, the SDH makes specific predictions about shifts in defensive strategy allocation that can, theoretically, be biochemically quantified. Anti‐herbivory traits at the population level can be assessed in many ways, but because of the vast biochemical diversity in the plant kingdom (Kaur et al. 2022), it can be prohibitively difficult to query biochemical defenses and variation across hundreds or thousands of individual plants (Jack et al. 2019). To address this challenge, we focus on general, conserved inducible defense mechanisms that offer the potential to reflect evolutionary shifts in investments between constitutive and induced responses. Two key enzymes known to be expressed by *M. polymorpha* (Constabel and Yip 2000; Mithöfer and Boland 2012; Soffan et al. 2014; Taranto et al. 2017) and to be involved in inducible defenses are polyphenol oxidase (PPO) and peroxidase (POD), which are rapidly upregulated following herbivore damage and play crucial roles in producing defensive compounds (War et al. 2012). Together with total protein quantification, these enzyme measurements provide complementary evidence of inducible defense responses, allowing us to detect changes that might not be captured by any single metric.

These theoretical frameworks have begun to be tested in the *M. polymorpha* system, providing an opportunity to examine defensive evolution in a well‐documented invasion. Previous empirical work investigating differences in *M. polymorpha* 's defenses against herbivory between ranges by Jack and Friesen (2019), found that an insect herbivore, the soybean looper ( *Chrysodeixis includens* ), a generalist that feeds on 174 plants across 39 families (Panizzi 2013), prefers native range *M. polymorpha* (an unfamiliar population) over familiar, invaded range genotypes. In other words, plants in the invaded range had become less palatable, suggesting that even a short co‐evolutionary history conferred more effective defense. However, this study did not aim to investigate changes to specific defensive mechanisms, such as changes in the production of digestion inhibitors and the production of toxins by *M. polymorpha* in response to co‐occurring or novel herbivores; such data could lend further evidence in support of the SDH by revealing evidence of a trade‐off between defensive types rather than a more broad reduction in specialized anti‐herbivore defenses.

**FIGURE 2:**
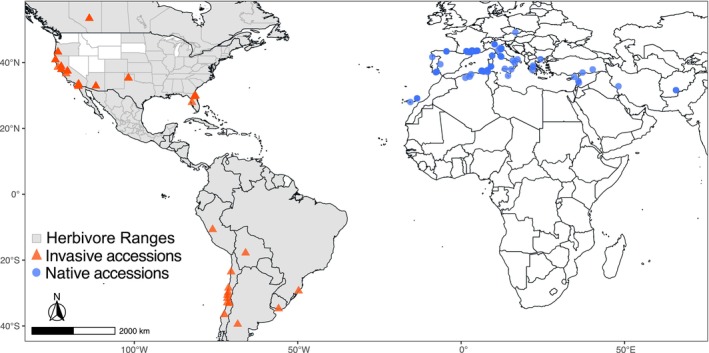
Geographic distribution of *M. polymorpha* accessions and herbivore ranges. The native range of *M. polymorpha* is shown in the Mediterranean region of Eurasia and North Africa by blue circles. The invaded range spans North and South America and points are shown with orange triangles. The ranges of the soybean looper and velvetbean caterpillar overlap with the invaded range and are shown as gray shaded areas in the Americas.

Our system presents a modified test of SDH predictions. Rather than testing newly arrived plants against novel herbivores, we test plants with 500 years of co‐evolution against herbivores from their invaded range. Thus, where SDH typically predicts reduced effectiveness against specialists in the invaded range, our system might show the opposite pattern due to this extended co‐evolutionary period. The experimental set‐up used by Jack and Friesen (2019) used Soybean Loopers to look for evidence of changes in host herbivory defenses between its native and invasive ranges. Here, we extend this system to further explore the changes in herbivory defense between the native and invaded ranges of *M. polymorpha* by adding a second herbivore, the Velvetbean Caterpillar ( *Anticarsia gemmatalis* ), a relative specialist that primarily consumes leguminous plants (Carner and Turnipseed 1977; Panizzi 2013) and tests of putative biochemical defenses. Because of issues around transatlantic insect importation, we use dietary breadth as a proxy for specialist vs. generalist selection pressures, recognizing this may not capture co‐evolutionary specificity (Ali and Agrawal 2012). The work presented here aims to test for rapid evolutionary changes in herbivore‐induced plant defenses using a natural experiment by comparing populations of *M. polymorpha* from its native and invaded ranges (Figure 2). Specifically, we investigate the following questions:

1. Are invaded range plants constitutively less palatable and better chemically defended against invaded range herbivores than native range plants?
2. Has selection in the invaded range altered the relative effectiveness of constitutive versus induced defenses against local herbivores?
3. Do constitutive and inducible defenses trade off? If so, do the effects of such a trade‐off give rise to differing preferences between generalist herbivores and more specialized herbivores?

This research aims to elucidate the evolutionary dynamics of herbivore defense mechanisms in *M. polymorpha* by leveraging a natural experiment to assess the interplay between host defenses and their impact on herbivore interactions.

## 2 Methods

### 2.1 *Medicago polymorpha* Accessions

*M. polymorpha* is an annual legume that is indigenous to the Mediterranean basin but has spread worldwide. It forms a mutualistic symbiosis with the rhizobium *Ensifer medicae* . Seeds for this study came from two sources (Table S1). First, a field collection of wild seeds gathered in 2015 across multiple sites in the native range. Pods were picked from the ground at least 1 m apart to ensure sampling distinct maternal lines. These lines were selfed for three generations in a common greenhouse to reduce maternal effects. Second, accessions from the USDA Germplasm Resources Information Network (GRIN) representing both native and invasive populations. Some accessions were selected from a field collection of wild plants collected between May and September of 2015. The field collection was created by collecting individual pods from the ground at least 1 m apart. These pods were assumed to represent unique maternal lineages because *M. polymorpha* is highly selfing in nature and the pods rarely disperse large distances (Lesins and Lesins 2012). All parental plants of the genotypes we used were started from a single pod, selfed from a single progeny for three generations, and grown in a greenhouse environment to control for maternal effects. Because evolutionary forces continue to act on these plants and because an initial accession may contain multiple genotypes after years of maintenance in a biobank, we use the term genotype here to reference the single paternal or maternal origin (Fu 2024). This method of developing a seed collection does not lead to artificial inbreeding in highly selfing plants such as *M. polymorpha* (Vitale et al. 1998). The remaining accessions were selected from the United States Department of Agriculture (USDA) Germplasm Resources Information Network (GRIN).

### 2.2 *Medicago polymorpha* Growth

*Medicago polymorpha* was grown under controlled conditions of 22°C/20°C (day/night) with 50% relative humidity, a 16‐h photoperiod, and light intensity set at 300 μmol/m^2^/s. These parameters were maintained to ensure uniform environmental conditions throughout the experiments. Two hundred seventy genotypes (123 from the invaded range and 190 from the native range; Table S1) chosen at random were used for the following experiments: herbivore preferences (constitutive defenses between ranges; within range constitutive vs. induced defenses), protein expression, peroxidase expression, and polyphenol oxidase expression.

Seeds were scarified with 600 grit sandpaper and planted into 158‐ml D‐pots (Stuewe & Sons, Tangent OR) filled with Sungro Sunshine Mix #1. Three seeds were planted into each cone 5 mm below the soil surface in the Washington State University, Vancouver greenhouse (45.732679, −122.635799) April 8–9, 2016. Plants were fertilized with slow‐release fertilizer (Osmocote Plus Outdoor & Indoor fertilizer pellets) and inoculated a week after planting with a rhizobium strain mixture of 10^7^ cells of equal parts *E. medicae* strain WSM419 and *E. meliloti* strain 1021. During germination, seeds were mist irrigated twice a day for 20 min and then mist‐irrigated daily as needed. The plants were grown for 8 weeks before use in the experiments.

An additional 36 genotypes (19 from the invaded range and 17 from the native range; Table S1) again chosen at random were used in a second set of experiments at Clark University: herbivore preference (induced defenses between ranges) and protein expression. Scarified seeds were imbibed for 24 h at 4°C and then germinated on 1.5% H_2_O agar plates in the dark for 24 h. Germinated seedlings were transferred to 158‐ml D‐pots containing pre‐moistened autoclaved Sungro Sunshine Mix #1. Plants were grown in an indoor growth chamber with the previously described settings and inoculum. Plants were watered with DI water three times a week. After 2 weeks, plants received a modified Hoagland's solution (Hoagland 1950) once a week (9 mM KCl, 5 mM CaCl_2_, 2 mM MgSO_4_, 4 mM NH_4_NO_3_, 1 mM KH_2_PO_4_, 1× standard micronutrient solution, and 200 μM Fe‐EDTA). Plants were harvested at 8 weeks. These conditions matched those in Washington state.

### 2.3 Herbivore Species

To assess variation in herbivory between populations of *M. polymorpha* from the two ranges of interest and to evaluate the effect of constitutive and induced resistance within each range, we selected two caterpillar species with differing dietary breadths. The more generalist species used was the larva of the Soybean Looper, *Chrysodeixis includens* (Lepidoptera: Noctuidae), known for its polyphagous nature, feeding on a wide variety of plants across multiple families (Herzog 1980). The more specialist species was the larva of the Velvetbean Caterpillar, *Anticarsia gemmatalis* (Lepidoptera: Noctuidae), which primarily consumes leguminous plants (Carner and Turnipseed 1977; Panizzi 2013). Third instar caterpillars, sourced from Benzon Research (Carlisle, PA), were reared on a multi‐species artificial diet from Southland Products Inc. before being used in the experiments. Due to regulatory restrictions, a caterpillar familiar to the native range of *M. polymorpha* was not used in our study. Larvae were contained, as required by our USDA plant pest permit (P526P‐15‐00942), in hard plastic deli boxes that come with lids that crimp on both sides of the box walls. The lids were sealed on three sides, but not the fourth to allow air exchange while preventing escape.

### 2.4 Biochemical Tissue Analysis

*Insect elicitor preparation*–24 h before tissue collection from experimental plants, a soybean looper and velvetbean caterpillar of approximately the same size were fed a mix of invasive and native range *M. polymorpha* leaf tissue. The day of the experiment, the larvae were decapitated and their heads were homogenized together with a mortar and pestle. The combined homogenate was mixed with 0.5 mL of DI water and kept on ice.

*Tissue collection*–95 genotypes were initially selected for the biochemical assays. To better control the induction of defenses, herbivory was simulated under consistent and controlled conditions. Scissors dipped in the insect homogenate were used to cut off a trifoliate (Time 0) and induce the production of herbivore chemical defense compounds. Trifoliates were similarly collected at 24 h. Each trifoliate was placed in a separate 96 deep well plate on ice (described in Soffan et al. 2014). Once all plants were sampled, the plates were flash frozen in liquid nitrogen and cryogenically stored at −80°C until analysis. 34 additional genotypes were collected for protein expression at Clark to ensure comparable induction for the additional preference test performed at that location.

*Extraction and leaf homogenization*–Frozen leaf tissue was quickly removed from tubes and weighed before thawing. The tissue was returned to tubes and again flash frozen in liquid nitrogen. The tissue was homogenized for 15 min at 300 rpm in a tissuelyzer (QIAGEN TissueLyser II) with a 2 mm steel bead. For each time point, two trifoliates were collected of each genotype in separate plates. Half of the tubes received 1 mL of 0.1% TCA buffer for enzyme analysis, while the other received 1 mL of PE buffer (1 mM EDTA, 88 mM Trizma Base, 10% glycerol) for total protein quantification (Jack et al. 2019). The tubes were centrifuged at 4°C for 10 min at 4500 rpm and the supernatant transferred into clean microcentrifuge tubes. The PE buffer tubes were then diluted to $\frac{1}{10}$ X.

*Protein Quantification, Peroxidase (POD) Activity, and Polyphenol Oxidase (PPO) Activity*–Protein quantification was performed using the Thermo Scientific PierceTM BCA Protein Assay Kit (Product number: 23337) according to manufacturer instructions for microplate samples. POD and PPO activity were measured following the protocol outlined in Jack et al. (2019).

### 2.5 Plant Preference Assays

#### 2.5.1 Constitutive Tissue Range Preference

We used 69 native and 69 invasive range *M. polymorpha* genotypes in unique pairings to assess herbivore preference for either native or invasive range tissue without any induced herbivory defenses. For each pair, a single trifoliate leaf from native range and invasive range genotypes was weighed and placed on opposite sides of a 95 mm petri dish divided down the center with a thin plastic ridge. One caterpillar was placed in the middle of the petri dish and left to feed for 24 h. After 24 h, the remaining leaf tissue was weighed to calculate how much of each genotype was eaten. Each genotype pairing was tested in two separate assays: one with the soybean looper and one with the velvetbean caterpillar.

#### 2.5.2 Induced Tissue Range Preference

We used 17 native and 17 invasive range *M. polymorpha* genotypes in unique pairings to assess preference for either native or invasive range tissue after induction of herbivory defenses. Plants were induced by simulating herbivory through a combined mechanical and chemical approach (Lin and Felton 2020), which has been shown to differ from host responses to damage alone (Steinbrenner et al. 2022; Prajapati et al. 2024). Leaf tissue was lacerated using sterilized medical scissors dipped in insect homogenate. Preference assays were conducted as above.

#### 2.5.3 Within Range Constitutive vs. Induced Defense Preference Assays

We used 40 native and 40 invasive range *M. polymorpha* genotypes to test the plasticity of herbivore preference. Within each range, half of the genotypes were induced using insect homogenate. After 24 h, the induced genotypes were paired with a different non‐induced genotype from the same range to assess preference for either induced or non‐induced (constitutive) leaf tissue. As above, tissue was placed on opposite sides of a petri dish with a caterpillar for 24 h and weighed before and after the feeding trial. Four pairs were removed from later analysis due to discrepancies between weights before and after the feeding trials.

### 2.6 Statistical Analysis and Mapping

#### 2.6.1 Software

The data were analyzed using R version 4.3.3 and lme4 1.1‐35.4 (Bates et al. 2015) or glmmTMB 1.1.11 (Brooks 2025) to fit linear and generalized linear mixed‐effect models, lmerTest 3.1‐3 (Kuznetsova et al. 2017) for *p*‐values, emmeans 1.10.2 (Searle et al. 1980; Lenth 2025) to get contrasts and post hoc comparisons, and cocor 1.1‐4 (Diedenhofen 2022) for testing the significance of differences between correlation coefficients, along with helper packages car 3.1‐2 (Fox et al. 2022), Hmisc 5.1‐1 (Harrell Jr 2025), RcmdrMisc 2.9‐1 (Fox and Marquez 2023), and broom 1.0.5 (Bolker and Robinson 2024). Normality and heteroskedasticity were checked using DHARMa 0.4.6 (Hartig 2024). Figures were created using the R packages tidyverse 2.0.0 (Wickham 2023), cowplot 1.1.3 (Wilke 2024), corrplot 0.92 (Wei and Simko 2024), plotrix 3.8‐4 (Lemon 2023), and patchwork 1.2.0 (Pedersen 2024). Points of origin for plant accessions were mapped using the R packages sf 1.0‐20 (Pebesma and Bivand 2023), rnaturalearth 1.0.1 (Massicotte and South 2023), rnaturalearthdata 1.0.0 (South et al. 2024), rnaturalearthhires 1.0.0.9000 (South et al. 2025), and ggspatial 1.1.9 (Dunnington 2023). Statistical results are summarized in Table 1 and model specification can be found at https://github.com/roweshaw/2025_Evidence_of_rapid_evolution_herbivory_trade_offs_m_polymorpha.git.

**TABLE 1:** Statistical analyses of plant‐herbivore interactions and defense mechanisms across native and invasive plant ranges. The table summarizes results (chi‐square, *t*‐tests, *F*‐tests) for effects related to results presented from this study.

Figure	Effect	Statistic	Estimate_OR	*p*	Significance
2					
	Plant range (native vs. invasive) [Main effect]	*χ* ^2^ (1) = 14.09	—	< 0.001	***
	Herbivore species (Looper vs. Velvet) [Main effect]	*χ* ^2^ (1) = 24.20	—	< 0.001	***
	Range × Herbivore [Interaction]	*χ* ^2^ (1) = 6.54	—	0.011	*
	Velvet caterpillar—preference for native tissue	*t* = 4.47	Δ proportion = 0.184 (±0.041)	< 0.001	***
	Soybean looper—preference for native tissue	*t* = 2.02	Δ proportion = 0.034 (±0.017)	0.045	*
3					
	Plant range (native vs. invasive) [Main effect]	*z* = −2.763	—	0.006	**
	Defense state (constitutive vs. induced) [Main effect]	*z* = −2.213	—	0.027	*
	Range × Defense [Interaction]	*z* = 3.892	—	< 0.001	***
	Soybean looper (native range)	*z* = −2.009	OR = 0.37	0.036	*
	Velvetbean caterpillar (invasive range)	*z* = 2.764	OR = 2.62	0.006	**
	Velvetbean caterpillar (native range)	*z* = −3.320	OR = 0.33	< 0.001	***
4					
	Plant range (native vs. invasive) [Figure 4 main effect]	*F* (1, 91.9) = 12.33	—	< 0.001	***
	Defense induction (before vs. after) [Figure 4 main effect]	*F* (1, 743.2) = 148.78	—	< 0.001	***
	Range × Defense [Figure 4 interaction]	*F* (1, 743.2) = 9.70	—	0.002	**
	Invasive range: constitutive vs. induced [Figure 4 post hoc]	*t* = 8.44	Δ (log protein) = 0.789 (±0.094)	< 0.001	***
	Native range: constitutive vs. induced [Figure 4 post hoc]	*t* = 10.77	Δ (log protein) = 0.468 (±0.043)	< 0.001	***
	Range × Defense [Figure 4 interaction]	*F* (1, 755.6) = 2.64	—	0.072	.
	Defense induction [Figure 4 main effect]	*F* (1, 757.7) = 23.32	—	< 0.001	***
	Plant range (native vs. invasive) [Figure 4 main effect]	*F* (1, 95.6) = 3.04	—	0.084	.
5					
	Total protein (trade‐off, overall) [Kendall's *τ*]	*z* = −13.01	*τ* = −0.560	< 0.001	***
	POD activity (trade‐off, overall) [Kendall's *τ*]	*z* = −5.02	*τ* = −0.219	< 0.001	***
	PPO activity (trade‐off, overall) [Kendall's *τ*]	*z* = −11.44	*τ* = −0.497	< 0.001	***
	Between‐range difference in trade‐off (Fisher's *z* test) [Figure 5A]	*z* = 1.76	—	0.078	.

*Note:* Significance symbols: *p* < 0 0.1 .; *p* < 0.05 *; *p* < 0.01 **; *p* < 0.001 ***.

Abbreviations: OR, odds ratio; POD, peroxidase; PPO, polyphenol oxidase.

#### 2.6.2 Preference Assays

**FIGURE 3:**
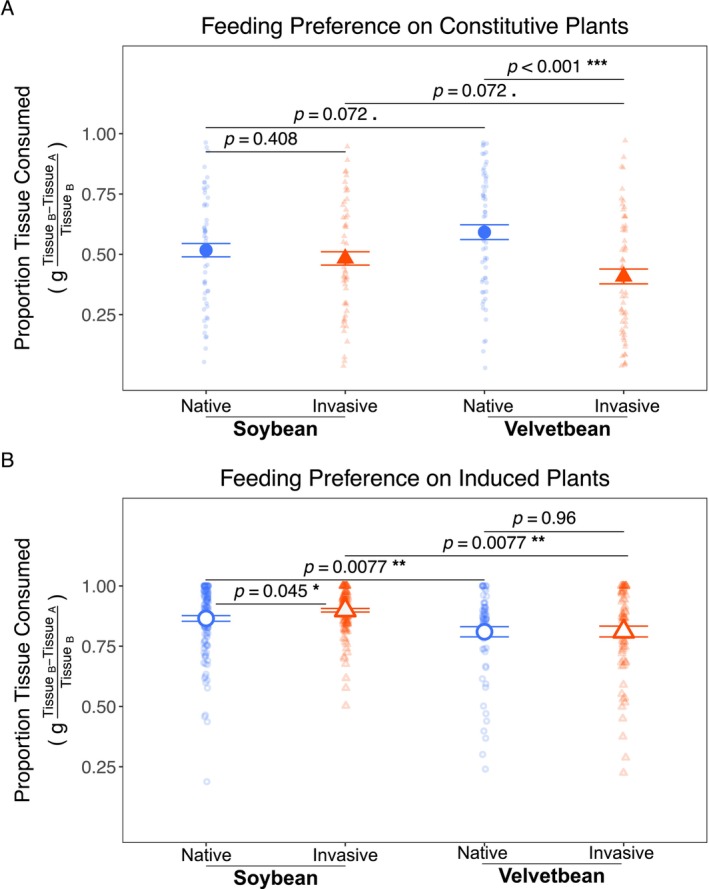
Herbivore feeding preferences between native and invasive *M. polymorpha* ranges. Herbivore preference is shown as the proportion of tissue consumed from each range relative to total tissue consumption $\left(g\;\frac{\left(\text{TissueB}-\text{TissueA}\;\right)}{\text{TissueB}}\right)$. (A) Feeding preference assays on constitutive (uninduced) tissues. Soybean Loopers showed no significant preference between ranges (*p* = 0.41), while Velvetbean Caterpillars significantly preferred native range tissue (*p* < 0.001 ***). (B) Feeding preference assays on herbivore‐induced tissues. Soybean Loopers showed a slight preference for invasive tissue (*p* = 0.045 *), while Velvetbean Caterpillars showed no preference (*p* = 0.96). Native values are shown as blue circles and invasive values are shown as orange triangles. Constitutive tissues are denoted by closed data points (A) and induced are denoted by open data points (B). Data were analyzed with a generalized linear mixed‐effect model with Type II Wald chi‐squared post hoc tests for significance. Bars display the means ± standard error. Additional statistical results can be found in Table 1. Significance symbols: *p* < 0.1 .; *p* < 0.05 *; *p* < 0.01 **; *p* < 0.001 ***.

**FIGURE 4:**
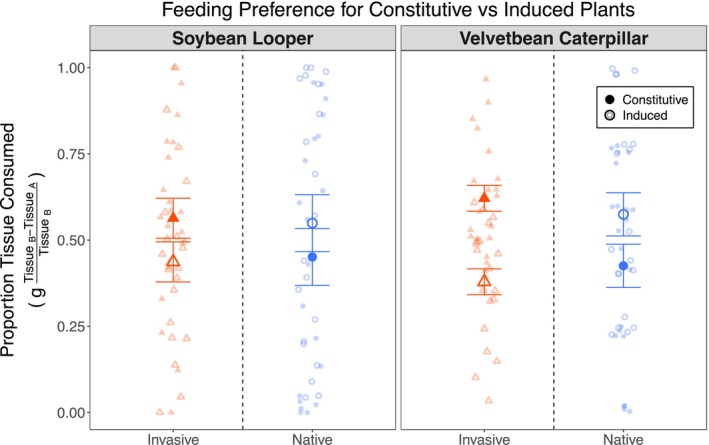
Herbivore feeding preferences between constitutive and induced tissues within *M. polymorpha* ranges. Herbivore preference is shown as the proportion of tissue consumed from each range relative to total tissue consumption $\left(g\;\frac{\left(\text{TissueB}-\text{TissueA}\;\right)}{\text{TissueB}}\right)$. Values for each pairings are shown for each group. The soybean looper and velvetbean caterpillar were given a choice between constitutive and induced *M. polymorpha* tissues from both ranges. Left panels: Soybean loopers showed no significant preference in invasive tissues (*p* = 0.41) but preferred induced over constitutive tissue in native plants (*p* = 0.036 *). Right panels: Velvetbean caterpillars strongly preferred constitutive over induced tissue in invasive plants (*p* = 0.006 **) but preferred induced over constitutive tissue in native plants (*p* < 0.001 ***). Native values are shown as blue circles, invasive values as orange triangles, constitutive tissues are closed data points, and induced tissues are open data points. Data were analyzed with a beta‐regression generalized linear mixed‐effect model (logit link) including an observation‐level random effect, with Type II Wald chi‐squared tests for significance. Bars display the means ± standard error. g, grams; OR, odds ratio. Additional statistical results can be found in Table 1. Significance symbols: *p* < 0.1 .; *p* < 0.05 *; *p* < 0.01 **; *p* < 0.001 ***.

Herbivore preference was quantified as the amount of tissue of the focal sample that was consumed divided by the total amount of tissue. Higher values indicate a stronger preference for the focal sample. A generalized linear model with a beta distribution was used to analyze the data. Pair was used as a random effect with range (Figure 3) and defense (Figure 4) as fixed effects.

#### 2.6.3 Biochemical Assays

Data were analyzed using a linear mixed model with Range as a fixed effect and Genotype as a random effect. The response variable for each assay was log‐transformed to bring the residuals closer to normal.

#### 2.6.4 Defensive Compound Production Correlations

Values from the biochemical assays at each time point were mean standardized to give a normal approximation and tested for statistically significant Pearson correlations after adjusting for multiple comparisons using Holm's method.

#### 2.6.5 Constitutive Expression vs. Inducibility Trade‐Offs

For each biochemical assay, we used Kendall's Tau on untransformed data to look for statistically significant monotonic correlations between constitutive expression and degree of inducibility, the difference between measured values at 0 and 24 h. We analyzed each range separately and as a combined dataset. To determine if the correlations between ranges were different from each other, the data were natural log transformed and analyzed using Fisher's *Z*‐test. To verify that the correlations between the variables were not spurious, since they were calculated from the same underlying data, we used an R implementation of the simulation procedure proposed by Morris et al. (2006) (Morris et al. 2006).

## 3 Results

Herbivore preferences indicated invasive genotypes were less palatable than native genotypes without prior defense induction and that preference changes upon induction of defenses. Overall, herbivore preference for invasive tissues versus native tissues was different (*p* < 0.001 ***) when testing plants with constitutive levels of defense expression (Figure 3A). This observed effect seemed to be driven by the Velvetbean Caterpillar, who demonstrated a strong preference for native range *M. polymorpha* tissues (*p* < 0.001 ***) while Soybean Loopers appeared to have no noticeable tissue preference (*p* = 0.41). Once herbivory defenses were induced, the Velvetbean preference disappeared (*p =* 0.96), while the Soybean Loopers demonstrated a slight preference for the invaded range tissue (*p* = 0.045 *). Unlike the constitutive preference tests, once defenses are induced, there is a difference in the overall feeding behaviors of the two herbivores (*p <* 0.001 ***) with the Velvetbean Caterpillars consuming less of both ranges compared to the Soybean Looper (Figure 3B).

Herbivores demonstrated greater sensitivity to induced defenses from the invaded range, indicating a difference in defense effectiveness between ranges. When given a choice between leaf tissues with constitutive or herbivore‐induced defenses, both herbivores showed a preference for constitutive tissues from invasive range genotypes, although it was only significant for Velvetbean Caterpillars (*p* = 0.006**) and not Soybean Loopers (*p* = 0.41). Interestingly, the inverse was true for the native range, where both demonstrated a significant preference for induced tissues (*p* < 0.001***). This effect was again stronger for the Velvetbean Caterpillars (*p* < 0.001***) than the Soybean Loopers (*p* = 0.036*; Figure 4).

**FIGURE 5:**
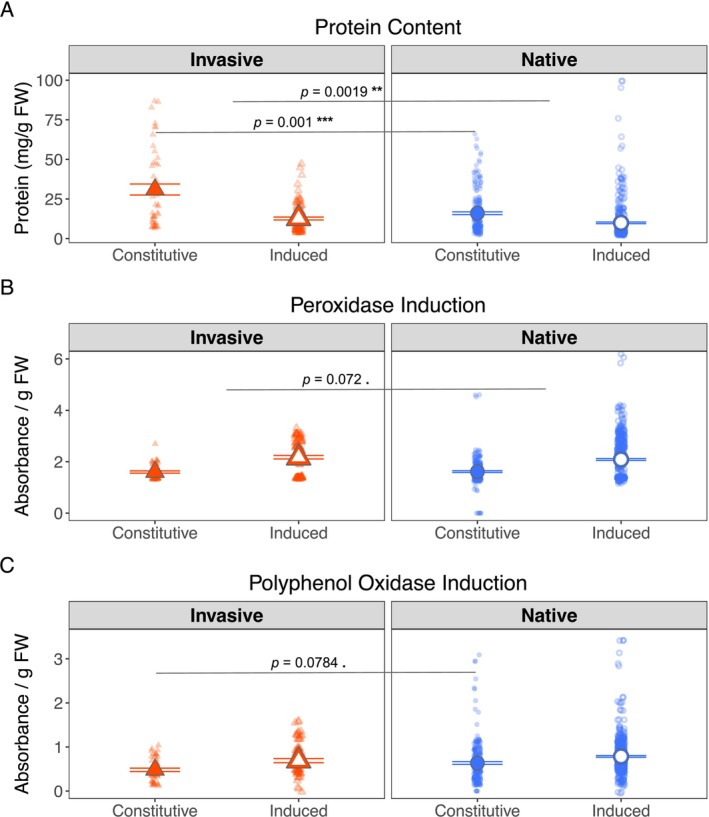
Biochemical markers associated with herbivore defense before and after induction in *M. polymorpha* . (A) Total protein content (mg protein g^−1^ fresh weight) differed by Range (*p* < 0.001 ***), Defense (*p* < 0.001 ***), and their interaction (*p* = 0.002 **). Post hoc contrasts revealed higher constitutive than induced protein in both invasive (*p* < 0.001 ***) and native (*p* < 0.001 ***) tissues. (B) Peroxidase (POD) abundance (absorbance g^−1^ FW) and (C) Polyphenol oxidase (PPO) abundance (absorbance g^−1^ FW) varied by Defense (*p* < 0.001 ***). Bars show means ± standard error. Native values are shown as blue circles, invasive values as orange triangles, constitutive tissues are closed data points, and induced tissues are open data points. Data were analyzed by linear mixed‐effects models (protein responses were log‐transformed) with Type III ANOVA. *n* = 94, g, grams; FW, fresh weight. Additional statistical results can be found in Table 1. Significance symbols: *p* < 0.1 .; *p* < 0.05 *; *p* < 0.01 **; *p* < 0.001 ***.

Herbivore preference is aligned with differences in protein content but not measured POD or PPO levels. Genotypes from the native and invaded ranges produced different amounts of protein when averaged over defense states (*p <* 0.001 ***) and both produced lower protein concentrations after induction (both *p* = < 0.001 ***). We further found evidence that the magnitude of change in total protein content after defense induction was different between ranges (*p* = 0.002 **) which aligns with apparent differences in herbivore preferences described previously (Figure 5A). As expected, genotypes from both ranges increased production of POD and PPO after induction (Figure 4) with no constitutive range differences. However, though they didn't meet the criteria for statistical significance, the magnitude of POD induction (*p* = 0.0718) and the absolute level of constitutive PPO both showed a marginal range‐dependent effect (*p =* 0.0783.).

**FIGURE 6:**
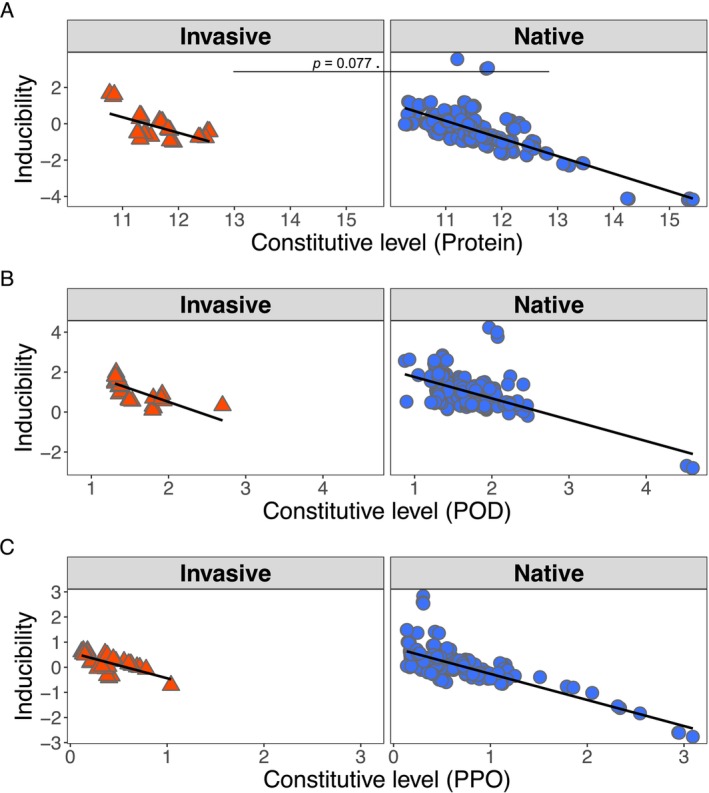
Trade‐offs between constitutive expression and inducibility of biochemical responses in *M. polymorpha* . Inducibility (log difference between constitutive and induced) is plotted against log‐transformed constitutive expression level (*n* = 94), faceted by range. The trade‐off was significant within ranges and overall for all 3 tested responses (A) Protein overall: *τ* = −0.560 (*p* < 0.001 ***). (B) Peroxidase (POD)overall: *τ* = −0.219 (*p* < 0.001 ***) (C) Polyphenol oxidase (PPO) overall: *τ* = −0.497 (*p* < 0.001 ***). Fisher's *z* tests (Zou's 95% CIs) showed a marginal range‐dependent difference in correlations for protein (*p* = 0.0777) and no significance for POD (*p* = 0.1989) or PPO (*p* = 0.3319). Native values are shown as blue circles and invasive values as orange triangles. Points denote genotype‐mean values; black lines are linear fits and data were analyzed with Kendall's rank correlation and Fisher's *z* tests with Zou's confidence intervals. Additional statistical results can be found in Table 1. Significance symbols: *p* < 0.1 .; *p* < 0.05 *; *p* < 0.01 **; *p* < 0.001 ***.

Constitutive expression and inducibility of measured defense responses were negatively correlated, indicating a trade‐off that was conserved between ranges. To complement the among‐range SDH comparisons above, we also examined trade‐offs within each range at the genotype level by testing the correlations between constitutive and inducible defenses with the goal of understanding potential trade‐offs between level of investment in constitutive defenses and the strength of inducible defenses and how they might vary between different plant ranges (Figure 6). We found a negative correlation between constitutive levels and inducibility upon herbivore attack for soluble protein levels (Figure 6A, *r*_invasive_ = −0.5873, *r*_native_ = −0.7691, *p* < 0.001 ***), POD (Figure 6B, *r*_invasive_ = −0.7486, *r*_native_ = −0.6159, *p* = < 0.001 ***), and PPO (Figure 6C, *r*_invasive_ = −0.6781, *r*_native_ = −0.768, *p* = < 0.001 ***). The correlations between ranges demonstrated a trend toward significance in the degree of the trade‐off for total protein content (*p* = 0.0777), suggesting that the observed trade‐off between the presence of constitutive defenses and the degree to which defenses can be induced is conserved in both plant ranges.

## 4 Discussion

The evolutionary dynamics of plant defenses following biological invasions represent opportunities to test our understanding of rapid adaptations under novel selection pressures. Hypotheses like the Enemy Release Hypothesis (ERH) and Shifting Defense Hypothesis (SDH) make distinct predictions about how invasive plants adapt to novel herbivore communities; however, empirical evidence for these frameworks remains limited. Jack and Friesen (2019) documented herbivore preference for unfamiliar native *M. polymorpha* compared to familiar invaded range tissues without prior defense activation, which suggested evolutionary changes that affect tissue palatability. Our work extends this framework by investigating the specific mechanisms underlying the documented preferences and characterizing shifts in constitutive and induced defense strategies that are central to SDH predictions. Importantly, our comparison tests evolutionary familiarity versus naivety rather than a simple native–invasive contrast; native range populations represent evolutionarily naive populations encountering unfamiliar herbivores, while invaded range populations have had centuries of co‐occurrence with New World herbivores. While some of our findings align with SDH predictions, others reveal unexpected patterns that illustrate the multifaceted nature of plant defense evolution during invasions (Callaway and Maron 2006).

The SDH predicts that defensive strategies of invasive plants will evolve in response to novel herbivore communities, resulting in an evolutionary divergence in defensive strategies between ranges. In line with this, between‐range tests of herbivore feeding preferences revealed a preference for familiar invaded range tissues over the unfamiliar native range tissues when both had only constitutively expressed defenses (Figure 3A), suggesting that invasive populations have more effective constitutive defenses against the tested herbivores than their native range counterparts. Though SDH predicts reduced effectiveness of constitutive defenses in invaded ranges, the coevolutionary history between host populations and herbivores is fundamental to understanding these predictions (Doorduin and Vrieling 2011). Since co‐occurrence of invaded range populations and the New World herbivores has spanned nearly 5 centuries, this result may represent an interesting snapshot in the coevolutionary trajectory of these organisms. The complete disappearance of the preference for native range tissues when defenses were induced, coupled with the Soybean Looper's slight preference for invasive over native induced tissues (Figure 3B), suggests that the effectiveness of induced defensive strategies has also diverged between ranges. The evolution of both defense types provides strong evidence for ongoing evolutionary adaptation in the invaded range. The fact that both defense types show evidence of evolutionary change suggests that selection pressures from the New World herbivores have been sufficiently strong to drive complex changes in *M. polymorpha* 's defensive strategy over the ~500 years since introduction.

Our findings extend traditional SDH predictions by demonstrating the importance of shared coevolutionary time in driving defense effectiveness. Classical SDH applications are often applied in simple range tests that assume a lack of coevolutionary adaptation between invaded range populations and herbivores of the invaded environment. However, our results suggest that sufficient time (~500 years) can allow for meaningful adaptation to novel environmental factors in invaded ranges such as enhanced baseline defenses against familiar New World herbivores compared to evolutionarily naive range populations (Thiel et al. 2020). This suggests that SDH predictions should account for invasion age and the duration of plant–herbivore co‐occurrence, recognizing that long‐established invasions may involve important secondary coevolutionary relationships rather than simple enemy escape scenarios in relative isolation.

Within‐range preference patterns additionally lend evidence in support of SDH predicted defense strategy evolution by revealing differential responses by both herbivore species, though these observations also suggest complex dynamics not captured by this study. The clear flip in herbivores' preference between the native and invaded ranges suggests there has been a relative shift in defense allocation over the course of the invasion (Figure 4), rather than a simple reduction in defense investment as predicted by the ERH. The consistency of the preference reversal across both herbivores suggests that this pattern reflects broader evolutionary changes rather than specialist‐specific responses (Figure 3). These within‐range patterns likely also reflect an ongoing coevolutionary arms race dynamic between plants and herbivores, where both plants and herbivores have been evolving in response to one another (Jack et al. 2019; Lloyd et al. 2025). Preference assays provide valuable evidence for evolutionary divergence, but they cannot fully disentangle plant‐specific adaptations from herbivore‐specific adaptations. Though we were unable to investigate the feeding preferences of herbivores from the Eurasian native range, the conserved flip in feeding preferences strongly suggests a divergence in the defense strategies of the two *M. polymorpha* ranges.

Our findings extend those presented by Jack and Friesen (2019), who first documented that soybean loopers prefer *M. polymorpha* genotypes from the native range over those from the invaded range. Like Jack and Friesen, we detected an overall herbivore preference for native range genotypes over those from the invaded range when testing constitutive defenses. However, when herbivore preference was examined for each species individually, this preference was only significant for Velvetbean Caterpillars and not for Soybean Loopers alone (Figure 4). This result deviates from the previous findings by Jack and Friesen. Notably, our Soybean Looper tissue consumption mean results showed the same directional preference as those previously reported, suggesting a common underlying biological pattern consistent between both studies. The difference in statistical significance can likely be explained by differences in both the overall sets of genotypes used as well as differences in the plant growth stages. While Jack and Friesen used 6‐week‐old plants grown in Michigan (USA), our study used 8‐week‐old plants grown under different conditions in Washington (USA) and Massachusetts (USA). These methodological differences could easily affect the detectability of the already modest signals in the original study.

To begin exploring potential mechanisms that may underpin the observed evolutionary shifts in defense strategies, we characterized three well‐documented biochemical responses that are common for many plants (Agrell and Oleszek 2003; Agrawal 2011; Agrawal and Hastings 2012). As expected (Soffan et al. 2014), we observed a strong induction of POD (Figure 5B) and PPO (Figure 5C) upon simulated herbivory in both invasive and native tissues. The examination of differences in POD and PPO abundances showed no range‐dependent differences in the degree of induction; however, measurements of POD revealed a notable trend indicating a potential difference in the magnitude of induction between ranges, while measurements of PPO found a similar trend for differences in constitutive expression. The possible difference in PPO constitutive abundance between ranges (Figure 5C) represents a potential biochemical signature of adaptive evolution in response to different herbivore communities. PPO's role in reducing protein digestibility through quinone formation directly links to anti‐herbivore defense (Soffan et al. 2014; Taranto et al. 2017). The marginally differing patterns observed in constitutive PPO levels and the magnitude of POD induction between ranges suggest selective pressure may have shaped how these enzymes are deployed in constitutive versus induced responses.

Despite the lack of significance in our two enzymatic tests, we found a strong range effect when measuring foliar protein content (Figure 5A). Invasive genotypes maintain a higher protein content in their constitutive state than their native counterparts. Though both ranges showed a sharp decline in soluble protein content upon defense induction, we found that the drop was more pronounced for the invasives than the natives. The reduction suggests a reallocation of protein resources when a plant is threatened (Garcia et al. 2021). The elevated levels in the invasive range paired with the pronounced decline suggest that invasives may exhibit a stronger reallocation of protein toward induced defense mechanisms; regardless of the specifics, the result points to a changed strategy in the invasive genotypes in comparison to the native genotypes. This apparent strategic difference between populations reflects the fundamental prediction of SDH—that plants exposed to different herbivore communities will evolve different defense deployment strategies (Doorduin and Vrieling 2011). Invasive populations have apparently evolved to optimize the effectiveness of their constitutive defenses against local herbivores while maintaining the capacity for induced responses. This biochemical signature aligns perfectly with SDH predictions and distinguishes our findings from the straightforward release from enemies predicted by ERH.

It is noteworthy that the enzymatic responses we measured did not predict the herbivore feeding preferences observed in our experiments. While PPO and POD are frequently used as proxies for defensive capability in plant‐herbivore studies, our results demonstrate that changes in these biochemical pathways may operate independently from the observed differences seen in the preference assays. This suggests that other unmeasured compounds are responsible for the observed shifts we report here. It is also plausible that, because *Medicago* spp. are known to possess multiple forms of both POD and PPO enzymes (Wei and Simko 2024; War et al. 2012; Soffan et al. 2014), our measurement of general enzymatic activity would obscure any differences in relative abundance of enzyme isoforms that may have been selected for in the distinct environments of the two populations (Thipyapong et al. 2007; Cabiddu et al. 2013; Soffan et al. 2014; Kosová et al. 2021). Our analyses were additionally only conducted at early time points for the induction of herbivory responses. In many species, including some legumes, analysis of oxidative defense activity has found that expression of POD and PPO enzymes can peak anywhere between 1 and 5 days post‐herbivory (Lagrimini and Rothstein 1987; Constabel and Yip 2000; War et al. 2012; Soffan et al. 2014), though the authors are unaware of time characterization in *Medicago polymorpha*. Future efforts should aim to investigate a broader suite of biochemical responses over multiple time points to capture more differences.

Building on these absolute measures of putative defenses, we then asked whether genotypes that invest heavily in baseline defenses are constrained by their investment with a reduced capacity for induced defenses. While our herbivory assays compared populations between ranges, understanding within‐population trade‐offs reveals the fundamental constraints that shape evolutionary responses regardless of invasive history. Across all three measured markers, we found a strong, negative correlation between constitutive expression and inducibility of defenses (Figure 6); in other words, a genotype's higher baseline defenses directly trade off with its ability to boost its defenses when under threat. This trade‐off was equally present for invasive and native genotypes, demonstrating a deeply conserved allocation constraint that has similarly been documented in other plant species (Chakraborty et al. 2023). This conserved trade‐off pattern may be explained by a fundamental physiological constraint on resource costs associated with maintaining high levels of both defense types simultaneously, creating selection pressure for efficient allocation (Rasmann et al. 2015; Chakraborty et al. 2023). While interesting, this pattern does not serve as evidence in support of the SDH and is not predicted under the framework. Instead, it reveals that the coevolutionary adaptation we document (Figures 2 and 3) operates within these conserved physiological limits.

Under the SDH framework, the observed trade‐off may allow plants to fine‐tune their defense strategies in response to changing herbivore communities without necessarily altering total defense allocation. In the invaded range, the optimized balance appears to favor enhanced effectiveness of constitutive defenses against local herbivores, potentially reflecting adaptation to the predominant herbivores over nearly 500 years of cooccurrence. Our finding directly aligns with the theoretical emphasis on changes in herbivore pressures posited by the SDH and provides strong evidence that invasive species optimize their defensive strategies by balancing resource allocation between immediately available and induced responses. However, these changes appear to operate through defense pathways other than the ones directly measured in this study, where we showed conserved trade‐offs regardless of evolutionary history. Overall, our evidence for these altered defense strategies in invasive populations better aligns with SDH than ERH, suggesting that while *M. polymorpha* may have initially benefited from enemy release upon introduction to the Americas, subsequent generations have undergone selection for optimized defense allocation. This temporal progression from ecological advantage to evolutionary adaptation likely contributes to the successful establishment and continued spread of *M. polymorpha* throughout North and South America.

The SDH predicts that invasive plants will likely possess decreased defense effectiveness against specialist herbivores and increased defense effectiveness against generalist herbivores in their introduced range, where they experience release from coevolved specialists and face novel pressure from resident generalists (Zhang et al. 2018). Our result showing a shift in preference for constitutive leaf tissue in one range to induced leaf tissue in the other was the greatest indication of rapid evolution between the native and invaded ranges, supporting the SDH. Invading plant populations encounter a suite of novel biotic and abiotic stresses in their new environments that may apply different selective pressures than what they are subjected to in their home ranges. Rapid adaptation prompted by these interactions is likely to be key for successful spread after establishment. In plants especially, the presence or absence of co‐evolved herbivores can have a profound effect on survival probability short‐term and can lead to evolutionary changes in not only defensive compound production but also competitive ability long‐term. Our biogeographic comparison of *M. polymorpha* found that genotypes in the invaded range have undergone rapid evolutionary changes that make them less preferred over their native range conspecifics. Since we found that constitutive and inducible defense levels of the measured proteins were conserved between the ranges, we cannot rule out that there have been changes in traits not directly related to anti‐herbivore defense production, for example a change in nutritional status or plant structure (Morrison and Hay 2011; Ho and Pennings 2013). However, the herbivores' shift in preference between the overall levels of defense compounds produced constitutively and those induced after herbivore attack aligns with components of the SDH and is evidence of rapid evolution between native and invaded range populations of *M. polymorpha* .

The ability of invasive plants to rapidly evolve specialized defenses in response to novel herbivores highlights the dynamic nature of plant invasions. These adaptations may not only enhance the competitive ability of invasive species, but also alter the ecological interactions within invaded ecosystems, potentially leading to shifts in community composition and ecosystem function. While our study provides evidence of evolutionary shifts in plant palatability consistent with SDH predictions, future research could more directly test ERH predictions by measuring field herbivory rates in both native and invaded ranges, quantifying growth and reproductive trade‐offs with defense investment, and examining resource allocation patterns. These findings challenge the assumption that invasive species are universally released from herbivore pressure and demonstrate that *M. polymorpha* has evolved effective defenses against novel herbivore threats, contributing to its invasion success.

## Author Contributions

**Shawna L. Rowe:** data curation (lead), formal analysis (supporting), investigation (equal), visualization (equal), writing – original draft (lead), writing – review and editing (equal). **Zoie C. Lopez:** investigation (equal), writing – review and editing (supporting). **Danaka Ross:** investigation (supporting). **Cynthia Sackos:** investigation (supporting). **Stephanie S. Porter:** conceptualization (equal), data curation (equal), funding acquisition (equal), resources (equal), supervision (equal), writing – review and editing (equal). **Maren L. Friesen:** conceptualization (equal), funding acquisition (equal), supervision (equal), writing – review and editing (equal). **Chandra N. Jack:** conceptualization (equal), data curation (lead), formal analysis (lead), methodology (lead), project administration (equal), resources (supporting), supervision (equal), visualization (equal), writing – original draft (equal), writing – review and editing (supporting).

## Disclosure

The authors have nothing to report.

## Conflicts of Interest

The authors declare no conflicts of interest.

## Supporting information


**Table S1:** List of plant accessions and places of origin used in this study. Table includes columns for unique accession ID, geographic range (native or invasive), named location of geographic origin, GPS coordinates, source collection information, and experimental usage that corresponds with experiments listed in the manuscript. Each accession is uniquely identified and tracked across multiple experiments to ensure reproducibility and proper documentation of plant material lineage.

## Data Availability

Code, data, copies of figures, and copies of tables are available at: https://github.com/roweshaw/2025_Evidence_of_rapid_evolution_herbivory_trade_offs_m_polymorpha.git.
